# Dietary xylooligosaccharides modulate oxidative stress and pathogen resistance in growing rabbits

**DOI:** 10.1186/s40104-025-01268-9

**Published:** 2025-11-07

**Authors:** Aipeng Mao, Xiaoyan Peng, Junning Pu, Yanbin Chen, Qingyue Liu, Jingyi Cai, Hua Zhao, Gang Jia, Gang Tian

**Affiliations:** 1 Animal Nutrition Institute, Sichuan Agricultural University, Chengdu, Sichuan 611130 People’s Republic of China; 2 Key Laboratory of Animal Disease-resistant Nutrition, Ministry of Education, Ministry of Agriculture and Rural Affairs, Key Laboratory of Sichuan Province, Chengdu, Sichuan 611130 People’s Republic of China

**Keywords:** Bacterial community, Fiber, Growing rabbit, Nutrient digestibility, Xylooligosaccharides

## Abstract

**Background:**

Given the high incidence of gastrointestinal disorders in intensive rabbit production, we assessed the effects of graded levels of xylooligosaccharides (XOS) on growth performance, nutrient digestibility and intestinal health in growing rabbits.

**Methods:**

The 35-day-old weaned rabbits (889.41 ± 0.41 g) were randomly assigned to five dietary treatments (0, 0.2, 0.3, 0.4 or 0.5 g/kg XOS) and the trial lasted for 35 d.

**Results:**

The results revealed that linear trend responses of body weight (BW) to XOS on d 21 and 35 (*P* ≤ 0.05). During d 1–21, 0.2 g/kg XOS increased average daily feed intake (ADFI) while 0.5 g/kg improved feed conversion ratio (FCR) significantly (*P* ≤ 0.05). Weight gain rate (WGR) showed a linear trend, while FCR showed a quadratic response (*P* ≤ 0.05). Throughout the 35-d trial, 0.2 and 0.3 g/kg XOS enhanced ADFI, and 0.4 g/kg XOS improved FCR significantly, average daily gain (ADG) demonstrated linear dose-responsiveness, while WGR and FCR showed quadratic trends (*P* ≤ 0.05). Notably, 0.2 g/kg XOS elevated serum glutathione peroxidase (GSH-Px) activity and ileal secretory immunoglobulin A (sIgA) levels. Furthermore, 0.3, 0.4 and 0.5 g/kg XOS reduced jejunal malonaldehyde (MDA) content, 0.4 g/kg XOS decreased serum MDA, and 0.5 g/kg XOS elevated serum immunoglobulin M (IgM) significantly (*P* ≤ 0.05). 0.2, 0.4, 0.5 g/kg XOS improved the digestibility of crude fiber (CF), 0.2 and 0.4 g/kg XOS increased acid detergent fiber (ADF), and neutral detergent fiber (NDF) also increased among all treatments, although 0.5 g/kg XOS reduced cellulase activity significantly (*P* ≤ 0.05). Furthermore, graded levels of XOS significantly changed the relative abundance of specific bacteria, and 0.4 and 0.5 g/kg XOS enhanced the content of valeric acid significantly (*P* ≤ 0.05).

**Conclusions:**

In conclusion, dietary supplementation of XOS serves as an effective nutritional strategy to optimize bacterial community in the cecum, improve fiber digestion and valeric acid production, while enhances resistance to intestinal pathogen infection and oxidative stress in rabbit production.

**Supplementary Information:**

The online version contains supplementary material available at 10.1186/s40104-025-01268-9.

## Introduction

Xylooligosaccharides (XOS) are oligomeric carbohydrates composed of 2–12 D-xylose units linked by β-1,4 glycosidic bonds and purified from lignocellulosic biomass [1]. These prebiotic compounds are abundantly present in agricultural byproducts, which represents a sustainable and renewable feedstock for XOS production [2, 3]. Dietary XOS supplementation demonstrates multifaceted benefits on intestinal health and growth performance of monogastric animals [4]. In broilers, XOS administration improves intestinal tissue morphology, while selectively modulating gut microbiota composition—notably elevating *Bifidobacterium* populations while suppressing *Clostridium perfringens* colonization, ultimately improving immunity and growth performance [5, 6]. Concurrently, XOS increased plasma level of immunoglobulin A (IgA), tumor necrosis factor-*α* (TNF-*α*), Immunoglobulin M (IgM) and interleukin-2 (IL-2) as well as the cecal content of butyric acid and acetic acid, while linearly reducing plasma levels of glutamic-pyruvic transaminase (GPT), cholesterol (CHO), high density lipoprotein (HDL) and very low-density lipoprotein (VLDL) linearly, which improving their intestinal health and immunity of laying hens [7, 8]. XOS supplementation demonstrates dose-dependent modulatory effects on porcine intestinal ecosystems. At lower supplementation levels (0.01% XOS), histological analysis revealed significantly increased jejunal and ileal villus height, accompanied by enhanced microbial α diversity indices and elevated short chain fatty acid (SCFA) concentrations by selective enrichment of *Lactobacillus*, *Streptococcus*, *Bifidobacterium*, and *Turicibacter* populations [9]. 0.05% XOS supplementation also showed the optimization of intestinal microbiota and increase the metabolites such as butyric acid and propionic acid [10, 11]. Notably, 0.04% XOS supplementation exhibited systemic antioxidant capacity via increase serum total antioxidant capacity (T-AOC), superoxide dismutase (SOD), and catalase activities, whereas decrease lipid peroxidation marker malondialdehyde (MDA), and significantly enhanced growth performance indices such as body weight (BW) and average daily gain (ADG) of piglets, which has the potential to prevent post-weaning intestinal dysfunction and improve production performance in piglets [12]. As a functional prebiotic, XOS demonstrate dual-action benefits for animal health and production performance: (1) protecting the gastrointestinal tract against colonization by pathogenic microbiota, (2) enhancing livestock products [13]. The unique physicochemical profile of XOS—characterized by exceptional acid tolerance (pH 2.5–8.0), high mechanical and thermal stress stability (600 MPa and 100 °C)—makes it particularly suitable for pharmaceutical formulations and functional feed development [14, 15].

Rabbits, as hindgut fermenters and obligate herbivores, have evolved specialized digestive systems adapted to process high-fiber diets consisting primarily of grass, hay and fibrous weeds. The majority of gastrointestinal disorders observed in captive rabbit populations are associated with specific nutritional imbalances, including either lower fiber intake, higher protein or carbohydrate levels in their feed [16, 17]. The gastrointestinal system of rabbits exhibits exceptional fragility. Post-weaning challenges arise from underdeveloped gastrointestinal function and intensive farming practices that utilize the prepared pellet feeds, which frequently lead to nutrition-related gastrointestinal disorders in captive rabbits [17]. Previous studies have shown that XOS can enhance the in vitro proliferation of lactic acid bacteria isolated from rabbit intestine [18]. However, adding 7.5 g/L of XOS to drinking water impairs rabbit growth and reduces nitrogen and energy retention [19].

XOS confer multiple benefits on gut health and growth performance in pigs and chickens. However, research on XOS in rabbits remains relatively limited, and its precise role in modulating intestinal digestion and production performance of rabbits remains unclear. We hypothesize that XOS supplementation may mitigate gastrointestinal complications associated with captive rabbit farming by promoting the activity of microorganisms. To test this hypothesis, we conducted a comprehensive investigation by supplementing different levels of XOS to the feed of growing rabbits. We systematically analyzed the effects of XOS on intestinal digestion and immunity. Our research provides a theoretical reference for optimizing the application of XOS in the nutritional management of rabbits.

## Materials and methods

### Xylooligosaccharides

XOS are provided by Yibin Yatai Biotechnology Co., Ltd. (China) with a content of 35%.

### Experimental design and diets

This study performed a single-factor treatment design. A total of 150 mixed-sex New Zealand White rabbits (35 d old, body weight 889.41 ± 0.41 g) were randomly divided into 5 groups (*n* = 30 per group): CON (0 g/kg XOS), T1 (0.2 g/kg XOS), T2 (0.3 g/kg XOS), T3 (0.4 g/kg XOS), and T4 (0.5 g/kg XOS), all rabbits were housed in individual galvanized wire mesh cages (50 cm × 50 cm × 40 cm, length × width × height). During the experiment, all rabbits were fed twice a day with free access to feed and water. The entire experiment lasted for 35 d, and the health and death of animals was recorded daily.

According to previous studies, the experimental diet was formulated to meet the nutritional requirements of growing rabbits [20] and supplemented with 0, 0.2, 0.3, 0.4 and 0.5 g/kg XOS in the form of equivalent replacement of ball grinding bran in the basal diet [7, 11, 19]. All diets were pelleted at the feed mill of the experimental facility and stored in a dark and dry environment until the start of the trial. The basic diet composition and nutrient level are shown in Table 1.

**Table 1:** Composition and nutrient levels of diets (DM basis)

Ingredients	Content, %	Nutrient	Content, %^2^
Alfalfa meal	17.50	DE, MJ/kg	10.20
Corn	15.00	CP	15.58
Soybean meal	9.20	CF	15.79
Corn germ meal	9.05	NDF	33.84
Wheat bran	15.00	ADF	18.96
Wheat middlings	6.00	ADL	5.50
Soybean hull	10.00	ST	16.84
Rice bran	5.00	Ca	0.60
Rice bran and hull	9.90	TP	0.52
Soybean oil	1.40		
CaCO_3_	0.50		
NaCl	0.38		
L-Lys (≥ 98.5%)	0.01		
DL-Met (≥ 99%)	0.04		
L-Thr (≥ 98.5%)	0.02		
Premix^1^	1.00		
Total	100.00		

^1^Premix provided the following per kg of the diets: VA, 6,667 IU; VD, 1,333 IU; VE, 15 IU; VK_3_, 1 mg; VB_2_, 3 mg; VB_6_, 0.5 mg; VB_12_, 10 μg; biotin, 100 μg; niacin, 35 mg; pantothenic acid, 10 mg; choline chloride, 100 mg; Fe (FeSO_4_·H_2_O), 35 mg; Cu (CuSO_4_·5H_2_O), 6 mg; Zn (ZnSO_4_·H_2_O), 35 mg; Mn (MnSO_4_·H_2_O), 8 mg; I (KI), 0.4 mg; Co (CoCl_2_·6H_2_O), 0.3 mg; Se (Na_2_SeO_3_), 0.05 mg

^2^Nutrient levels were calculated values

### Sample collections

Feed samples were collected from each group according to the national standards of the People’s Republic of China GB/T 14699.1-2023 [21]. Fecal samples were collected from 10 animals in each group for 4 consecutive days (from d 22 to 25) and treated with 10% hydrochloric acid for nitrogen fixation, and the samples were mixed evenly and dried to constant weight, then crushed and screened 40 mesh.

On d 22 of the experiment, 6 rabbits in each group were selected for collect blood samples from the auricular vein, and the samples were then centrifuged to obtain serum. Then the rabbits (*n* = 6 per group) were slaughtered in an industrial slaughterhouse and the tissue of jejunal, the mucosa of ileum, the chyme of ileum and cecum were collected. All samples were stored at −80 °C for further analysis.

### Growth performance

During the experiment, feed intake and body weight were recorded weekly, and growth performance were calculated according to the following formula:

$$\text{Average daily gain}\;(\mathrm{ADG})\;=\;(\text{Final weight}-\text{Initial weight})/\mathrm{days}$$

$$\mathrm{Weight}\;\mathrm{gain}\;\mathrm{rate}\;(\mathrm{WGR})\:=\:(\mathrm{Final}\;\mathrm{weight}-\mathrm{Initial}\;\mathrm{weight})/\mathrm{Initial}\;\mathrm{weight}$$

$$\mathrm{Average}\;\mathrm{daily}\;\mathrm{feed}\;\mathrm{intake}\;(\mathrm{ADFI})\:=\:\mathrm{Total}\;\mathrm{feed}\;\mathrm{intake}/\mathrm{days}$$

$$\mathrm{Feed}\;\mathrm{conversion}\;\mathrm{rate}\;(\mathrm{FCR})\:=\:\mathrm{Total}\;\mathrm{feed}\;\mathrm{intake}/(\mathrm{Final}\;\mathrm{weight}-\mathrm{Initial}\;\mathrm{weight})$$

### Serum biochemical indices

The concentrations of total protein (TP), albumin (ALB), globulin (GLB), total cholesterol (TC), triglyceride (TG), alanine aminotransferase (ALT), aspartate aminotransferase (AST), alkaline phosphatase (ALP), glucose **(**GLU**)** and urea in serum were determined using the fully automatic biochemistry analyzer 3100 (Hitachi, Japan), and the kits used in the analyzer were obtained from Maccura Biotechnology Co., Ltd. (Chengdu, China).

### The antioxidation of serum and jejunal tissues

The activity of superoxide dismutase (SOD), glutathione peroxidase (GSH-Px) and the content of malondialdehyde (MDA) in serum and jejunal tissue were determined using commercial assay kits (Nanjing Jiancheng Bioengineering Institute, Nanjing, China), and all procedures were conducted following the provided instruction manual.

### The immunity of serum and ileal mucosa

The levels of immunoglobulin A (IgA), immunoglobulin G (IgG), immunoglobulin M (IgM) in serum and the levels of secretory immunoglobulin A (sIgA), IgM and mucin 2 (MUC2) in ileum were measured using the corresponding ELISA kits (Jiangsu Meimian Industrial Co., Ltd., Yancheng, China) according to the manufacturer’s instructions.

### Apparent digestibility of nutrients

The contents of gross energy (GE) in fecal and diet samples were measured following to ISO 9831-1998 [22], the contents of dry matter (DM), crude protein (CP), ether extract (EE), crude fiber (CF), neutral detergent fiber (NDF), acid detergent lignin (ADL) and acid insoluble ash (AIA) in all samples was measured according to the national standards of the People’s Republic of China GB/T 6435-2014 [23], GB/T 6432-2018 [24], GB/T 6433-2006 [25], GB/T 6434-2022 [26], GB/T 20806-2022 [27], GB/T 20805-2006 [28] and GB/T 23742-2009 [29], respectively, and acid detergent fiber (ADF) was measured by the agricultural industry standard of the People’s Republic of China NY/T 1459-2022 [30]. AIA was used as an endogenous indicator and the apparent digestibility of nutrients were calculated according to the following formula:

Apparent digestibility of nutrients (%) = [1 − (the content of AIA in the diets/the content of AIA in the fecal) × (the content of a nutrient in the fecal/the content of a nutrient in the diets)] × 100.

### The activity of enzymes in the digestive tract

The enzymatic activities of amylase, lipase, and trypsin in the jejunum, as well as cellulase, hemicellulase, and pectinase in the cecum were quantified using commercial assay kits (Nanjing Jiancheng Bioengineering Institute, Nanjing, China).

### Analysis of short chain fatty acids

The 3 g of cecal digesta were homogenized with distilled water (1:1, w/v), vortexed, and centrifuged at 12,000 × *g* for 10 min. 1 mL of supernatant was mixed with 0.2 mL of 25% metaphosphoric acid, incubated for 30 min, and centrifuged again under the same conditions. Subsequently, a 100 μL of the resulting supernatant was combined with 100 μL methanol, vortexed, centrifuged (12,000 × *g*, 10 min), and the final supernatant stored at −20 °C. Short chain fatty acid concentrations were determined using a VARIAN CP-3800 gas chromatograph (Agilent Technologies, USA).

### Analysis of bacterial community in cecum

Total genomic DNA of cecum contents was extracted and verified by 1% agarose gel electrophoresis. PCR amplification was performed using forward primer (5'-ACTCCTACGGGAGGCAGCAG-3') and reverse primer (5'-GGACTACHVGGGTWTCTAAT-3') targeting the V3–V4 region of the 16S rRNA gene. Sequencing was conducted on the Illumina NovaSeq PE250 platform. Bioinformatic processing included: (1) Quality control of raw sequencing data using fastp (version 0.20.0); (2) Read assembly using FLASH (version 1.2.7); (3) OTU clustering at 97% similarity threshold and chimeric sequence removal with UPARSE (version 7.1); and (4) Taxonomic annotation using RDP classifier (version 2.2) against the SILVA 16S rRNA database (version 138) with a 70% alignment confidence threshold.

### Statistical analysis

Data are expressed as mean ± standard error and analyzed using one-way ANOVA through SPSS 27.0 (SPSS Inc., Chicago, IL, USA) for statistical computation and GraphPad Prism 10.1.2 for graphical representation. Differences between CON and each treatment groups were assessed using Dunnett's multiple comparison test. Additionally, orthogonal polynomial contrast analyses (linear and quadratic) were applied to growth performance data to analyze dose-response trends across XOS supplementation levels. β-Diversity analysis was performed using Bray-Curtis distances with Adonis-based PERMANOVA. Bacterial community and physiological indices correlations were evaluated via Pearson's rank correlation coefficients. *P* ≤ 0.05 was denoted as different significantly. Network relationships were visualized using R 4.2.2.

## Results

### Effects of XOS on survival rate and growth performance in growing rabbits

**Fig. 1:**
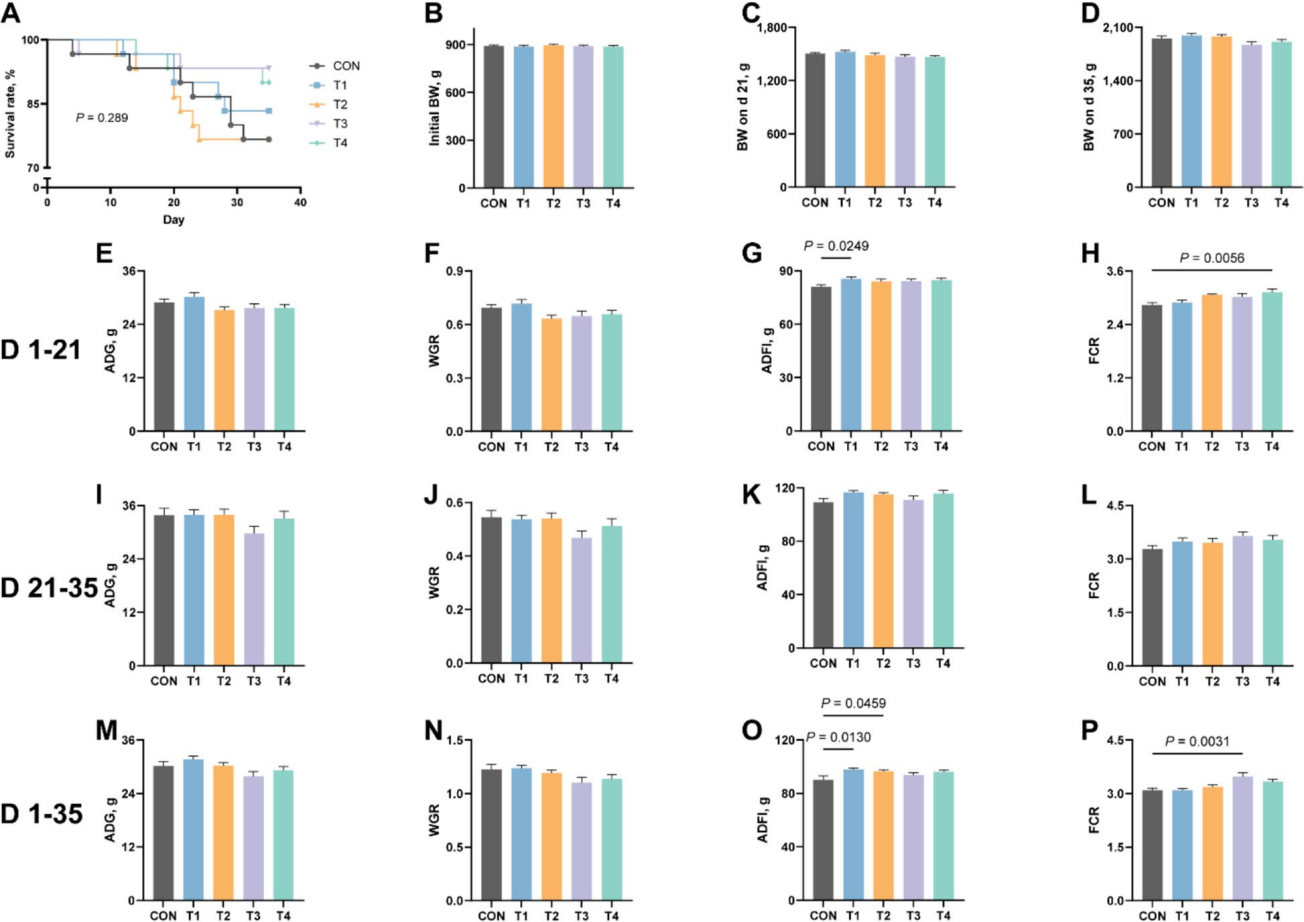
Effects of XOS on survival rate and growth performance in growing rabbits. **A** The survival rate of growing rabbits. **B**–**D** BW on d 0, d 21 and d 35 in growing rabbits. **E**–**H** ADG, WGR, ADFI and FCR on d 1–21 in growing rabbits. **I**–**L** ADG, WGR, ADFI and FCR on d 21–35 in growing rabbit. **M**–**P**. ADG, WGR, ADFI and FCR on d 1–35 in growing rabbits. BW, body weight; ADG, average daily gain; WGR, weight gain rate; ADFI, average daily feed intake; FCR, feed conversion ratio

As illustrated in Fig. 1, dietary supplementation with XOS demonstrated a tendency to enhance the survival rate of experimental animals throughout the trial period, although this improvement did not significance (*P* > 0.05, Fig. 1A). Notably, BW on d 22 and 35 of the experimental period exhibited linear responses to XOS levels (*P* ≤ 0.05, Additional file 1). During the initial phase (1–21 d), significant enhancements were observed in ADFI for the T1 group (Fig. 1G) and FCR for the T4 group (*P* ≤ 0.05, Fig. 1H). WGR demonstrated a linear progression while FCR showed a quadratic pattern in relation to XOS levels (*P* ≤ 0.05, Additional file 1). Throughout the entire experimental period, XOS improved ADFI in both T1 and T2 groups, and enhanced FCR in the T3 group significantly (*P* ≤ 0.05, Fig. 1O and P). ADG exhibited a linear dose-response relationship to XOS levels, while both WGR and FCR displayed a quadratic trend (*P* ≤ 0.05, Additional file 1). However, during the later experimental phase (21–35 d), no significant treatment effects were detected for ADG, WGR, ADFI or FCR (*P* > 0.05). These findings collectively suggest that XOS supplementation may enhance survival rate and promote feed consumption in rabbits.

### Effects of XOS on serum biochemical indices in growing rabbits

As presented in Table 2, the supplementation of XOS in growing rabbit diets demonstrated no significant effects on protein such as TP, ALB, GLB or lipid metabolism parameters (TC and TG) in serum (*P* > 0.05). Furthermore, the enzymatic activities of ALT and ALP remained unaffected by XOS administration (*P* > 0.05). Compared to the CON group, AST activities also did not differ significantly according to Dunnett's multiple comparison test. Additionally, no statistically significant alterations were observed in serum GLU and Urea concentrations following XOS supplementation (*P* > 0.05).

**Table 2:** Effects of XOS on serum biochemical indices in growing rabbits

Items	Groups					*P*-value
	**CON**	**T1**	**T2**	**T3**	**T4**	
TP, g/L	48.64 ± 2.47	50.98 ± 2.40	50.46 ± 1.16	46.70 ± 2.61	46.14 ± 1.19	0.404
ALB, g/L	34.74 ± 1.84	35.22 ± 1.82	35.89 ± 0.62	32.42 ± 1.55	33.65 ± 0.81	0.551
GLB, g/L	12.89 ± 0.45	15.76 ± 1.31	13.81 ± 0.82	14.28 ± 1.65	12.95 ± 0.41	0.347
TC, mmol/L	1.70 ± 0.41	1.71 ± 0.12	1.83 ± 0.07	1.53 ± 0.16	1.55 ± 0.09	0.386
TG, mmol/L	1.28 ± 0.25	1.09 ± 0.10	1.05 ± 0.11	1.22 ± 0.16	0.97 ± 0.11	0.683
ALT, U/L	23.91 ± 3.25	32.10 ± 3.81	23.20 ± 0.57	29.96 ± 4.84	29.09 ± 3.62	0.404
AST, U/L	9.19 ± 2.65	17.59 ± 5.04	8.31 ± 1.45	8.66 ± 1.08	4.74 ± 0.25	0.048
ALP, U/L	172.40 ± 9.30	163.67 ± 12.37	197.00 ± 9.77	190.20 ± 28.76	172.67 ± 10.72	0.494
GLU, mmol/L	6.68 ± 0.22	7.07 ± 0.26	6.90 ± 0.10	6.52 ± 0.23	6.65 ± 0.22	0.439
Urea, mmol/L	3.55 ± 0.23	4.05 ± 0.34	3.45 ± 0.23	3.25 ± 0.25	3.02 ± 0.25	0.101

Multiple comparisons were analyzed using Dunnett's test

### Effects of XOS on antioxidant indices in serum and jejunal tissue of growing rabbits

**Fig. 2:**
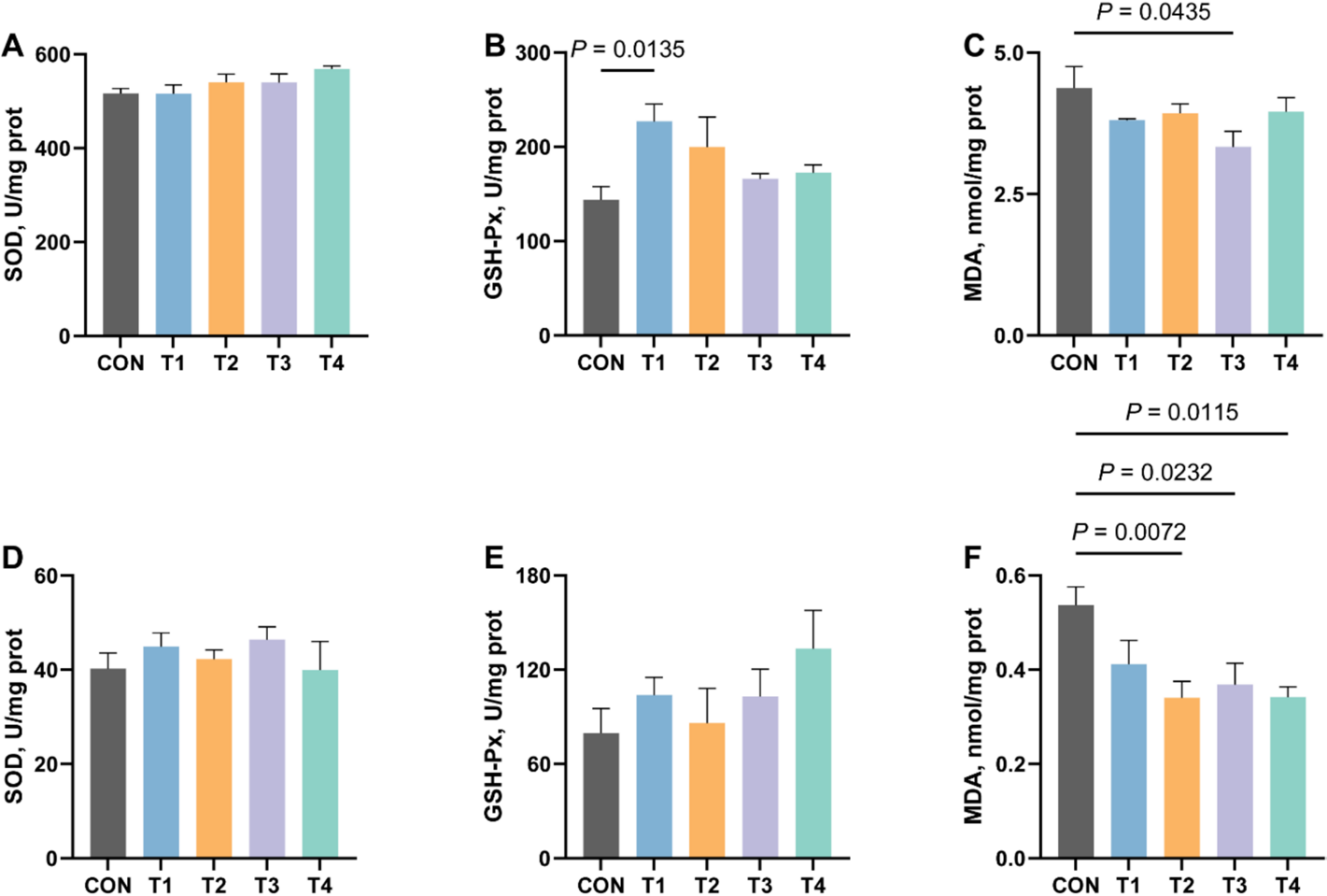
Effects of XOS on antioxidant indices in serum and jejunum of growing rabbits. **A** The activity of SOD in serum of growing rabbits. **B** The activity of GSH-Px in serum of growing rabbits. **C** The content of MDA in serum of growing rabbits. **D** The activity of SOD in jejunum of growing rabbits. **E** The activity of GSH-Px in jejunum of growing rabbits. **F** The content of MDA in jejunum of growing rabbits. SOD, superoxide dismutase; GSH-Px, glutathione peroxidase; MDA, malondialdehyde

As illustrated in Fig. 2, dietary supplementation with XOS modulated antioxidant capacity in both serum and jejunal tissue of growing rabbits. Although XOS administration showed no significant alteration in SOD activity, it exhibited a significant enhancement of serum GSH-Px levels in T1 group compared to the CON group (*P* ≤ 0.05, Fig. 2B). The most prominent antioxidative effect of XOS was observed in its capacity to reduce the content of MDA, comparative analysis revealed that jejunal tissue MDA levels were significantly decreased in T2, T3 and T4 groups compared to the CON group, while serum MDA concentrations showed statistically significant reduction in the T3 group (*P* ≤ 0.05, Fig. 2C and F).

### Effects of XOS on immune indices in serum and ileum of growing rabbits

**Fig. 3:**
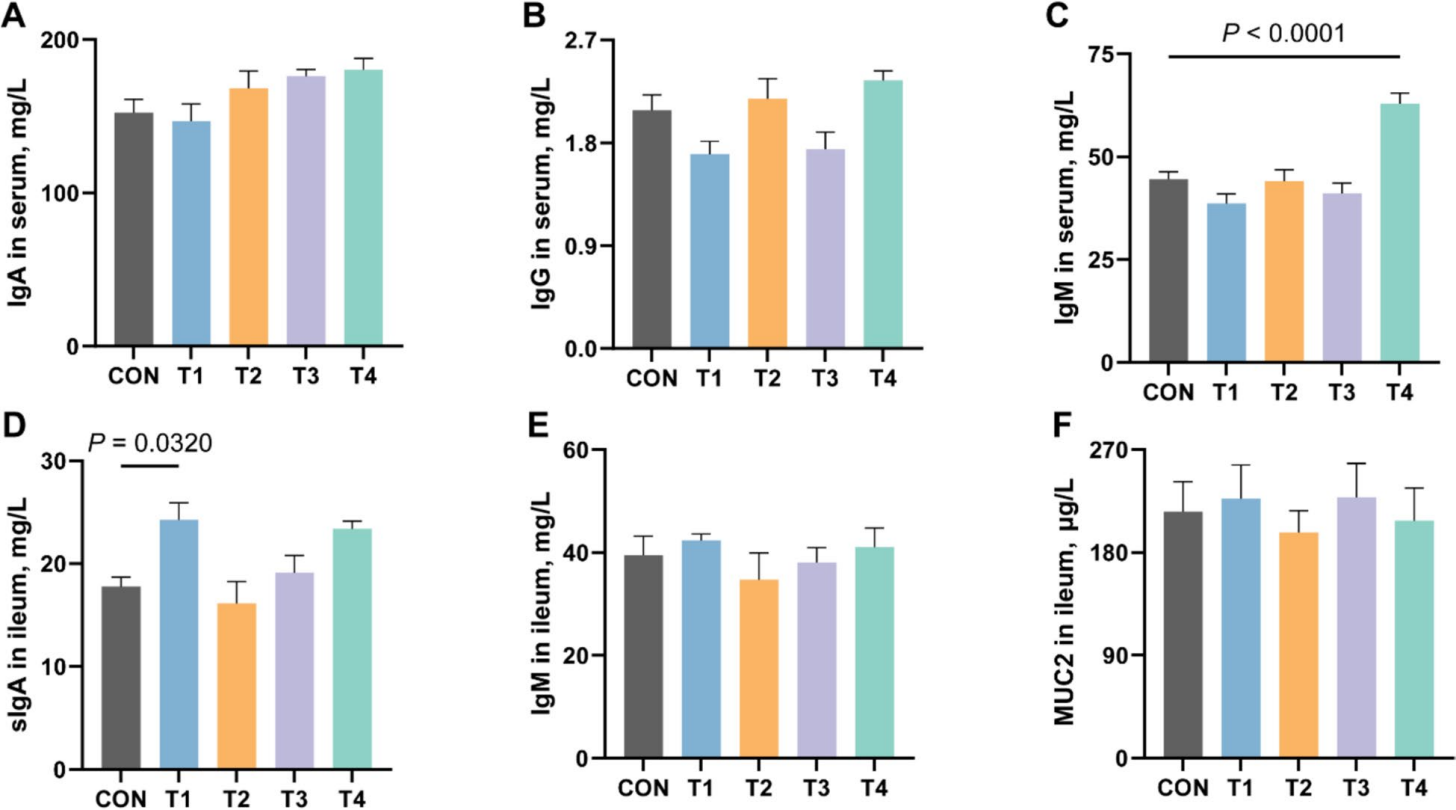
Effects of XOS on immune indices in serum and ileum of growing rabbits. **A** The content of IgA in serum of growing rabbits. **B** The content of IgG in serum of growing rabbits. **C** The content of IgM in serum of growing rabbits. **D** The content of sIgA in ileum of growing rabbits. **E** The content of IgM in ileum of growing rabbits. **F** The content of MUC2 in ileum of growing rabbits. IgA, immunoglobulin A; IgG, immunoglobulin G; IgM, immunoglobulin M; sIgA, secretory immunoglobulin A; MUC2, mucin 2

As depicted in Fig. 3, dietary supplementation with XOS elicited distinct immunomodulatory effects in growing rabbits. The T4 group demonstrated a significant increase in serum IgM levels compared to the CON group (*P* ≤ 0.05), while IgA and IgG concentrations remained unaffected in serum (*P* > 0.05). Notably, ileal analysis revealed marked enhancement of sIgA in the T1 group relative to the CON group (*P* ≤ 0.05). However, no statistically significant alterations were observed in ileal IgM levels or MUC2 expression among treatment groups (*P* > 0.05).

### Effects of XOS on apparent digestibility of nutrient in growing rabbits

**Fig. 4:**
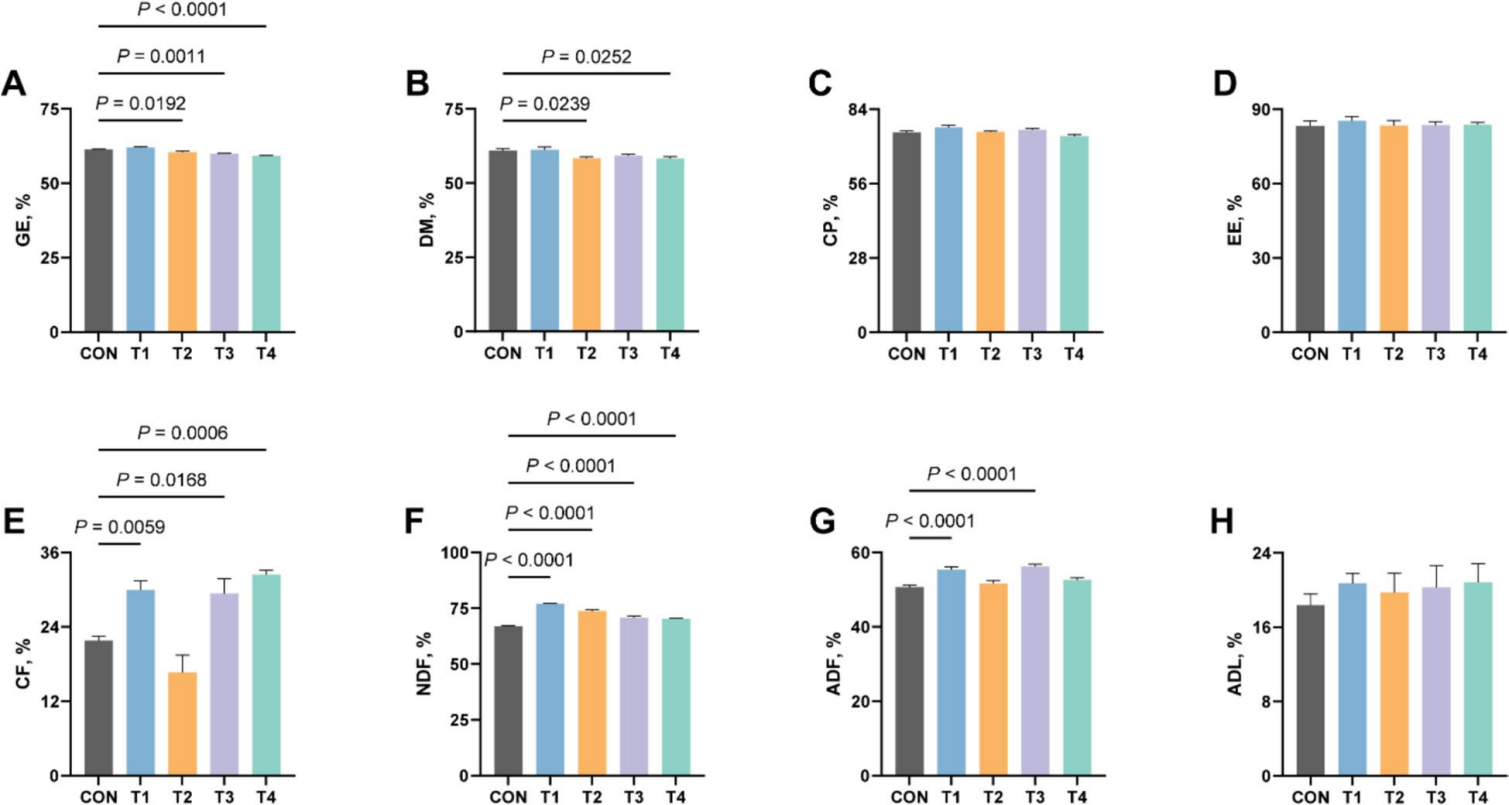
Effects of XOS on apparent digestibility of nutrient in growing rabbits. **A** The apparent digestibility of GE in growing rabbits. **B** The apparent digestibility of DM in growing rabbits. **C** The apparent digestibility of CP in growing rabbits. **D** The apparent digestibility of EE in growing rabbits. **E** The apparent digestibility of CF in growing rabbits. **F** The apparent digestibility of NDF in growing rabbits. **G** The apparent digestibility of ADF in growing rabbits. **H** The apparent digestibility of ADL in growing rabbits. GE, gross energy; DM, dry matter; CP, crude protein; EE, ether extract; CF, crude fiber; NDF, neutral detergent fiber; ADF, acid detergent fiber; ADL, acidic detergent lignin

To further investigate the reasons for XOS-induced enhancement in feed intake, we evaluated its effects on nutrient digestibility in rabbits (Fig. 4). The results revealed that XOS supplementation reduced the digestibility of energy in T2, T3 and T4 groups compared to the CON group (*P* ≤ 0.05), while DM digestibility was reduced in T2 and T4 groups significantly (*P* ≤ 0.05). More importantly, XOS significantly improved fiber utilization efficiency, CF notably increased in T1, T3 and T4 groups, ADF was enhanced in T1 and T3 groups, and NDF showed significant elevation among all treatment groups (*P* ≤ 0.05). These findings collectively indicate that XOS supplementation substantially modulates the digestibility of energy, DM and fiber, potentially explaining its feed intake-promoting effects via improved fiber digestibility.

### Effects of XOS on the activity of digestive enzymes in growing rabbits

**Fig. 5:**
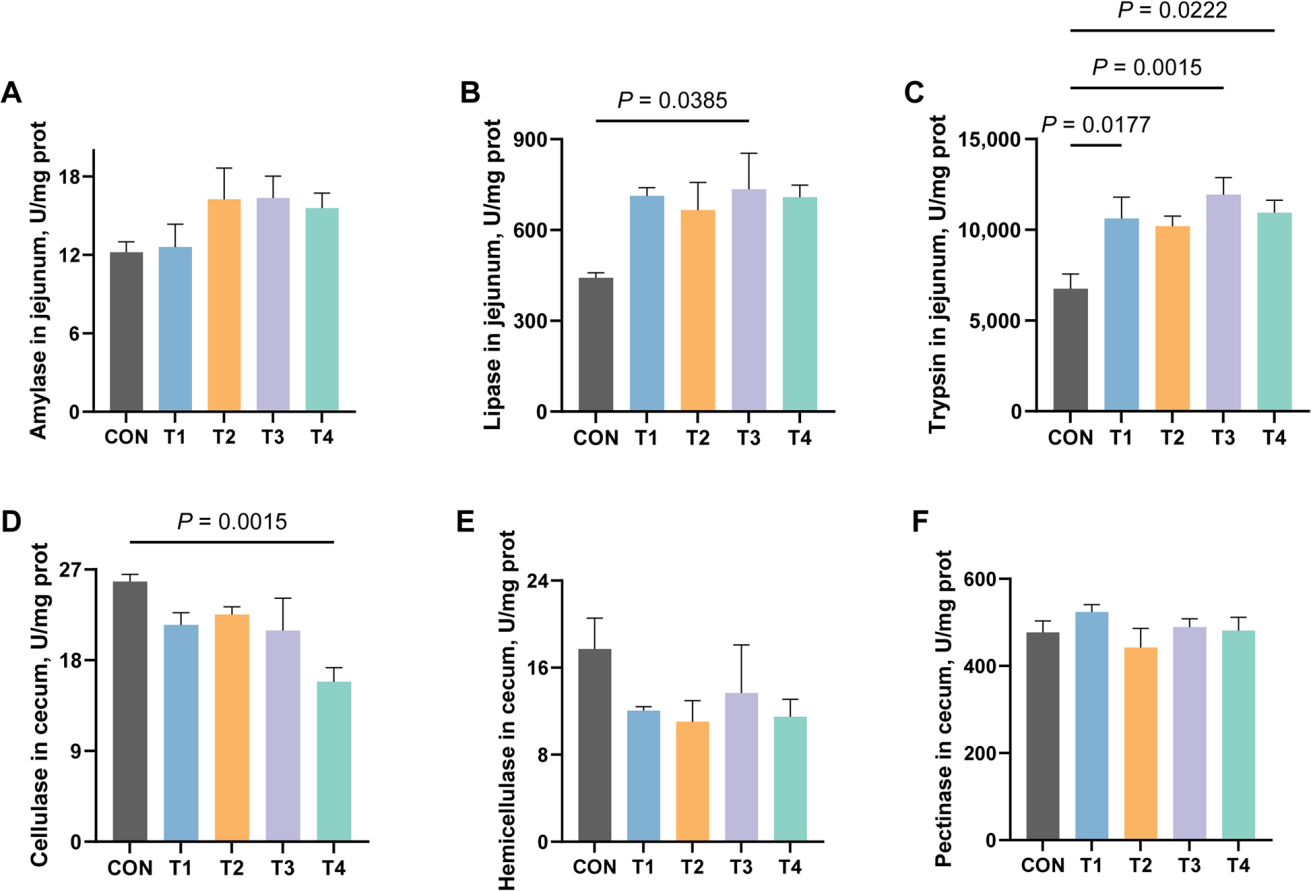
Effects of XOS on the activity of digestive enzymes in growing rabbits. **A** The activity of amylase in the jejunum of growing rabbits. **B** The activity of lipase in the jejunum of growing rabbits. **C** The activity of trypsin in the jejunum of growing rabbits. **D** The activity of cellulase in the caecum of growing rabbits. **E** The activity of hemicellulose in the caecum of growing rabbits. **F** The activity of pectinase in the caecum of growing rabbits

To further elucidate the mechanisms underlying the effects of XOS on nutrient digestion and absorption, we investigated its effects on digestive enzyme activities in the ileum and cecum (Fig. 5). The results demonstrated that lipase activity was significantly elevated in the T3 group compared to the CON group, while trypsin activity showed enhancement in T1, T3 and T4 groups significantly (*P* ≤ 0.05). However, cellulase activity in the cecum exhibited a significant reduction in the T4 group (*P* ≤ 0.05, Fig. 5D).

### Effects of XOS on the composition and diversity of bacterial community in cecum of growing rabbits

**Fig. 6:**
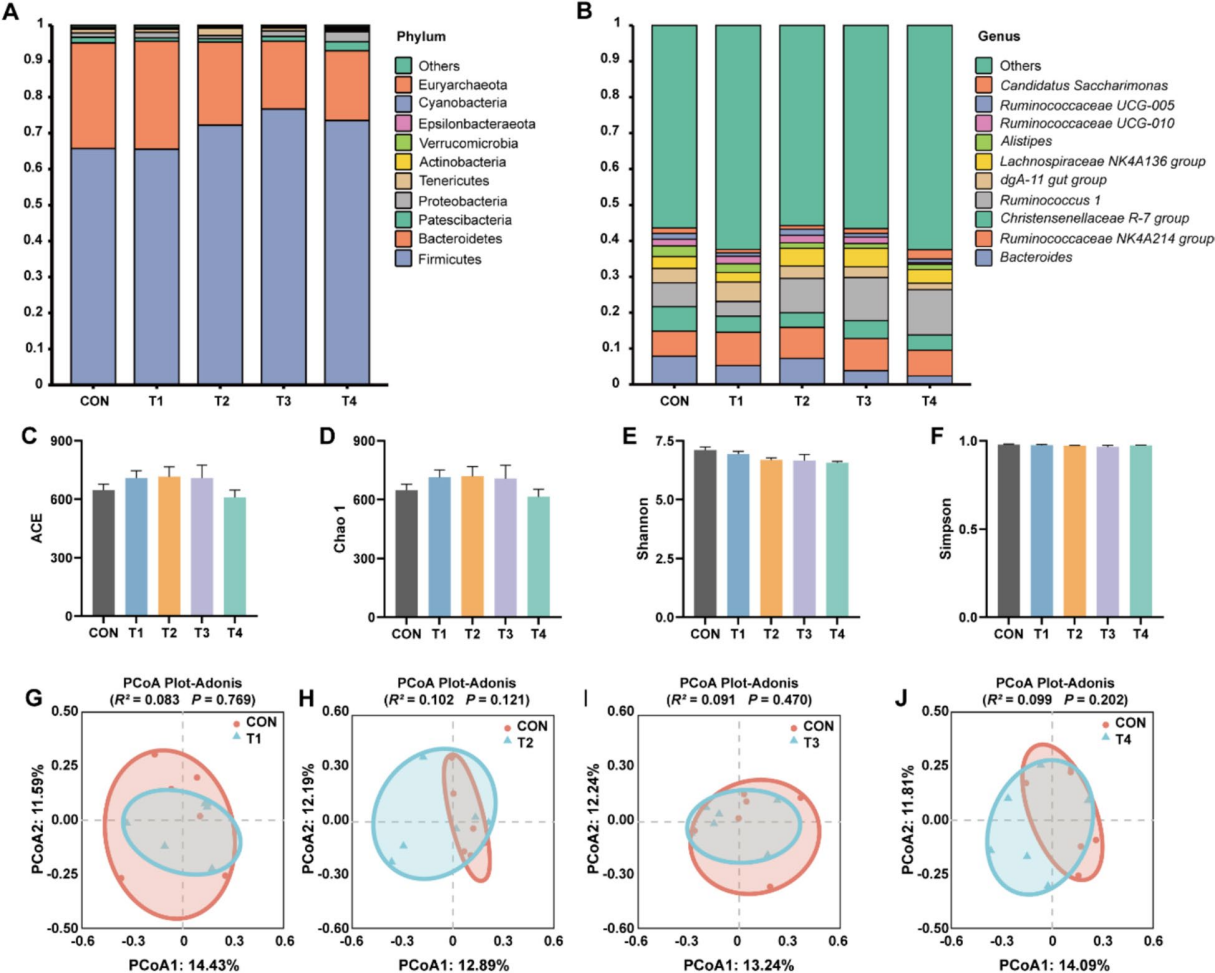
Effects of XOS on the composition and diversity of bacterial community in cecum of growing rabbits. **A** Bacterial community composition at the phylum level in the caecum of growing rabbits. **B** Bacterial community composition at the genus level in the caecum of growing rabbits. **C** ACE indices of α diversity in the caecum of growing rabbits. **D** Chao 1 indices of α diversity in the caecum of growing rabbits. **E** Shannon indices of α diversity in the caecum of growing rabbits. **F** Simpson indices of α diversity in the caecum of growing rabbits. **G** Comparison of β diversity between CON and T1 group. **H** Comparison of β diversity between CON and T2 group. **I** Comparison of β diversity between CON and T3 group. **J** Comparison of β diversity between CON and T4 group

Given that rabbits primarily digest fiber through microbial activity in the cecum, a process generating SCFAs. We further analyzed bacterial community and SCFA contents in cecum (Fig. 6 and Table 3). At the phylum level, Firmicutes and Bacteroidetes predominated across all groups (CON = 95.10%, T1 = 95.52%, T2 = 95.32%, T3 = 95.53%, T4 = 92.92%), followed by Patescibacteria and Proteobacteria in CON, T1, T3 and T4 groups (CON = 2.74%, T1 = 2.52%, T3 = 2.90%, T4 = 5.28%). However, Tenericutes emerged as the secondary dominant phylum in the T2 group (2.11%). At the genus level, *Bacteroides*, *Ruminococcaceae NK4A214 group*, *Christensenellaceae R-7 group* and *Ruminococcus 1* collectively dominated in the CON group (28.33%). The T1 group exhibited distinct dominance patterns with *Ruminococcaceae NK4A214 group*, *dgA-11 gut group* and *Bacteroides* constituting 19.99% of total abundance. Notably, *Ruminococcus 1* and *Ruminococcaceae NK4A214 group* sequentially prevailed as the most abundant genera in T2, T3 and T4 groups (T2 = 18.17%, T3 = 20.99%, T4 = 19.78%), demonstrating treatment-dependent variations in genus composition (Fig. 6B). The α diversity of bacterial community analysis revealed no significant difference in ACE, Chao 1, Shannon and Simpson indices among XOS-supplemented groups compared to the CON group (*P* > 0.05, Fig. 6C–F). Compared to the CON group, PCoA results also demonstrated no significant difference based on Bray-Curtis distances in T1 (Adonis: *R*^2^ = 0.083, *P* = 0.769), T2 (Adonis: *R*^2^ = 0.102, *P* = 0.121), T3 (Adonis: *R*^2^ = 0.091, *P* = 0.470) and T4 (Adonis: *R*^2^ = 0.099, *P* = 0.202) groups, respectively (*P* > 0.05, Fig. 6G–J). These findings suggest that XOS supplementation did not broadly reshape bacterial community in cecum of growing rabbits.

**Table 3:** Effects of XOS on short chain fatty acids in cecum of growing rabbits

Items	Groups					*P*-value
	**CON**	**T1**	**T2**	**T3**	**T4**	
Acetic acid, mg/g	1.67 ± 0.16	1.28 ± 0.16	1.74 ± 0.31	1.84 ± 0.19	2.21 ± 0.10	0.058
Propionic acid, mg/g	0.19 ± 0.01	0.19 ± 0.05	0.17 ± 0.02	0.21 ± 0.02	0.23 ± 0.01	0.750
Butyric acid, mg/g	0.38 ± 0.07	0.25 ± 0.04	0.39 ± 0.09	0.36 ± 0.04	0.47 ± 0.02	0.216
Valeric acid, mg/g	0.11 ± 0.01	0.13 ± 0.01	0.13 ± 0.02	0.15 ± 0.01^*^	0.17 ± 0.00^*^	0.004

Multiple comparisons were analyzed using Dunnett's test, with asterisks (*) denoting significant differences between each treatment group and the CON group

### Effects of XOS on short chain fatty acids in cecum of growing rabbits

As shown in Table 3, compared to the CON group, XOS supplementation did not demonstrate significant effects on the concentrations of acetic acids, propionic acids and butyric acids (*P* > 0.05). However, it enhanced valeric acid levels in the T3 and T4 groups significantly (*P* ≤ 0.05).

### Correlation analysis between different bacterial communities and physiological indices of growing rabbits

**Fig. 7:**
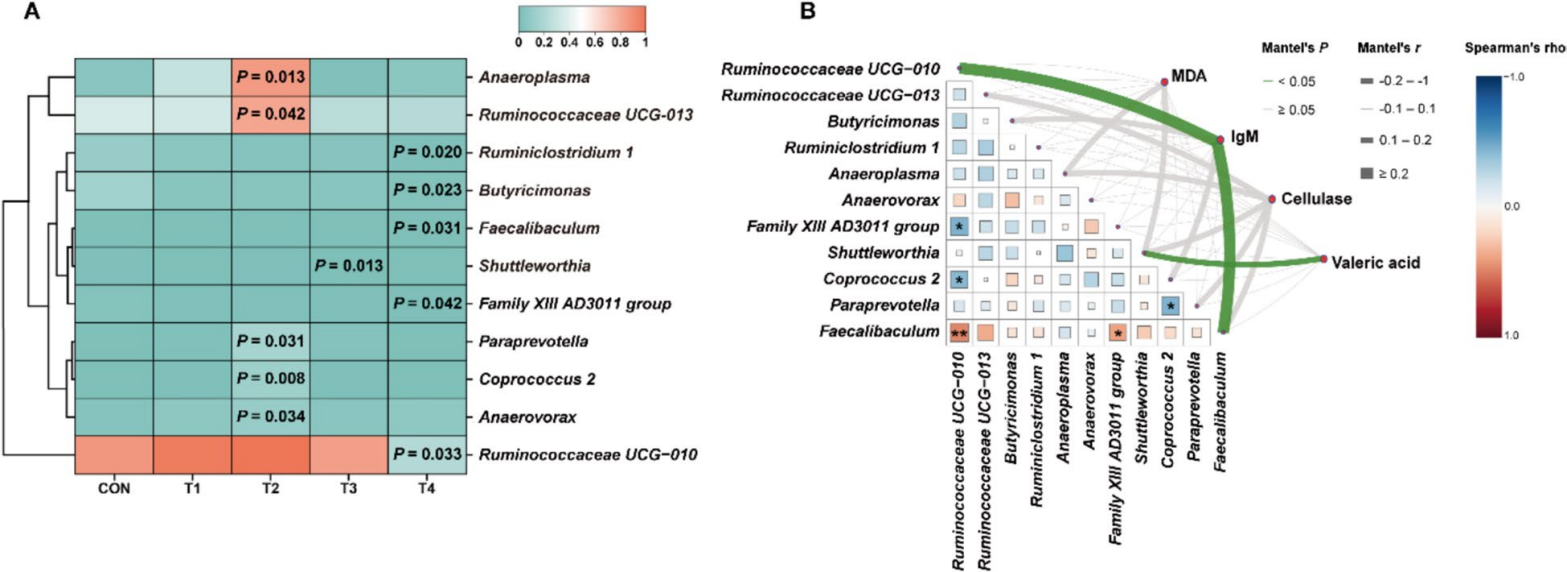
Comparison of differential genus and correlation analysis with physiological indicators. **A** Comparison of differential genus in cecum of growing rabbits. **B** Correlation analysis between bacteria and physiological indicators. *, ** indicate *P* ≤ 0.05, *P* ≤ 0.01, respectively. Spearman's correlation coefficients are donated with a color gradient. Physiological indicators were correlated with each bacterium by Mantel test. Edge width corresponds to the Mantel's *r* statistic for the corresponding distance correlations, and edge color denotes the statistical significance

To further investigate whether XOS supplementation could induce bacterial alterations, we analyzed differential bacterial composition at the genus level and identified 11 differentially genera (Fig. 7A). Specifically, compared to the CON group, the T2 group exhibited significant increases in relative abundance of *Ruminococcaceae UCG-013*, *Anaeroplasma*, *Anaerovorax* and *Paraprevotella* (*P* ≤ 0.05), with *Coprococcus 2* demonstrating a highly significant elevation (*P* ≤ 0.01). In contrast, the relative abundance of *Shuttleworthia* was significantly reduced in the T3 group (*P* ≤ 0.05), *Ruminococcaceae UCG-010*, *Butyricimonas*, *Ruminiclostridium 1*, *Family XIII AD3011 group* and *Faecalibaculum* showed marked decreases in the T4 group (*P* ≤ 0.05).

To further investigate the relationships between differential bacterial genera and host physiological indicators, Spearman correlation analysis was performed to identify potential bacterial interactions. The results revealed significant correlations between *Ruminococcaceae UCG-010* and both *Family XIII AD3011 group* and *Coprococcus 2*, *Family XIII AD3011 group* and *Faecalibaculum,* as well as *Paraprevotella* and *Coprococcus 2* (*P* ≤ 0.05). Notably, *Faecalibaculum* exhibited a highly significant correlation with *Ruminococcaceae UCG-010* (*P* ≤ 0.01). Subsequent Mantel test analysis identified significant correlations between specific bacteria and key physiological parameters: IgM levels demonstrated significant correlations with *Ruminococcaceae UCG-010* and *Faecalibaculum* (*P* ≤ 0.05), while valeric acid concentration showed a significant relationship with *Shuttleworthia* (*P* ≤ 0.05). These findings suggest potential functional linkages between differential general dynamics and critical physiological processes in the host organism.

## Discussion

XOS, as a functional prebiotic additive, have been widely incorporated into food and feed industry. They not only provide nutritional benefits but also enhance animal growth performance and disease resistance [31, 32]. Previous studies indicate that dietary supplementation with 0.2 g/kg XOS significantly improved ADG of broilers [33]. In piglet trials, 0.5 g/kg XOS markedly increased BW at 56 d of age. Furthermore, graded XOS levels exhibited quadratic effects on BW at 56 d, ADG and gain to feed ratio from d 28 to 56 [34]. In another study, XOS levels showed a linear negative correlation with FCR [35]. In the present study (d 1–21), 0.2 g/kg XOS significantly enhanced ADFI, whereas 0.5 g/kg XOS improved FCR. Notably, XOS supplementation showed linear effects on WGR and quadratic effects on FCR. Over the entire trial period (d 1–35), 0.2 and 0.3 g/kg XOS increased ADFI, while 0.4 g/kg XOS increased FCR throughout the experimental phase, WGR and FCR variations displayed quadratic responses, while ADG showed linear effects, and linear relationships were also observed between XOS levels and BW on d 21 and 35. Although 0.4 and 0.5 g/kg XOS improved survival rate in growing rabbits, the differences were not statistically significant. This aligns with findings from broiler studies where XOS supplementation at 0.1 g/kg failed to induce significant changes in ADG or BW [36], suggesting that subthreshold XOS levels may be insufficient to trigger measurable improvements in BW or survival rate.

Our findings indicate that XOS administration exerted no significant effects on serum biochemical parameters, similar observations have been reported in weaned piglets [9, 35]. Notably, our study revealed that XOS supplementation demonstrated certain antioxidant effects in both jejunal tissue and serum of growing rabbits. Oxidative stress, associated with various chronic conditions including cardiovascular diseases and malignancies, can be mitigated through lifestyle modifications or antioxidant supplementation [37]. The most extensively studied lipid peroxidation products include MDA, 4-hydroxy-nonenal (HNE) and F_2_-isoprostane 15(S)-8-iso-prostaglandin F_2α_ [38]. MDA, a terminal product of polyunsaturated fatty acid peroxidation, accumulates with increased free radical production [39]. Studies have demonstrated that MDA enhancing ROS generation by facilitating metmyoglobin formation and non-heme iron release, thereby promotes protein oxidation in rabbit [40]. The glutathione peroxidase family comprises four distinct mammalian selenoproteins that catalyze peroxide reduction using glutathione, providing protection against oxidative damage from dietary hydroperoxides and xenobiotic metabolism [41]. In our study, XOS supplementation at 0.2 g/kg significantly enhanced serum GSH-Px activity. Moreover, dosages of 0.3, 0.4, 0.5 g/kg XOS effectively reduced MDA content in jejunal tissue, with the 0.4 g/kg dose additionally demonstrating significant serum MDA reduction. These findings suggest that appropriate XOS supplementation enhances antioxidant capacity and confers protection against oxidative challenges from dietary components and environmental stressors.

Furthermore, our study demonstrated that dietary supplementation with 0.2 g/kg XOS significantly enhanced sIgA levels, while 0.5 g/kg XOS administration markedly increased serum IgM levels. Immunoglobulins execute their protective functions through coordinated interactions between variable and constant regions, enabling targeted neutralization and elimination of pathogenic microorganisms and toxins [42, 43]. Mechanistically, the distinctive C-terminal domain of IgA has been shown to inhibit influenza A and other sialic-acid-binding viruses [44], while simultaneously activating pathogen clearance through IgA Fc receptor (FcαRI/CD89) mediated phagocytic mechanisms [45, 46], these synergistic actions establish IgA as a critical defender against mucosal pathogen invasion [47, 48]. IgM contributes to immune homeostasis through two primary pathways: preventing autoimmune disorders via enhanced clearance of cellular debris [49, 50], and inhibiting microbial proliferation during early infection phases through viral/bacterial neutralization [51–53]. The XOS-induced elevation of sIgA and IgM observed in this investigation collectively provides robust protection against enteric toxins and pathogens.

To investigate the physiological effects of XOS in rabbit intestines, we analyzed changes in nutrient digestibility and digestive enzyme activity. The results demonstrated that dietary supplementation with 0.4 g/kg XOS significantly enhanced lipase activity, while 0.2, 0.4 and 0.5 g/kg XOS markedly increased trypsin activity. However, no improvement in the digestibility of EE or CP was observed with XOS administration. In addition, 0.5 g/kg XOS notably reduced cellulase activity without significantly affecting hemicellulase or pectinase activities, but XOS supplementation exhibited varying enhancement in the digestibility of CF, ADF and NDF paradoxically. These findings collectively suggest that XOS influences nutrient digestion and absorption, but that this effect may not be mediated through modulation of digestive enzyme activities in the intestinal tract of growing rabbits.

As herbivores, rabbits' digestion and absorption of nutrients depend not only on intestinal enzyme activity but also crucially on the functions of gut microbiota. The microbial diversity in rabbits is most abundant in the cecum and colon [54], and primarily consists of bacteria (10^11^/g) and yeasts (10^6^/g) in cecum [55]. Young rabbits predominantly harbor *Streptococcus* and *Enterobacteriaceae* in their lower gastrointestinal tract, while adult rabbits are dominated by *Bacteroides* dominance in the small intestine, cecum and colon, with composition influenced by age, diet and antibiotic [56]. Our bacterial analysis revealed that XOS did not extensively reshape the cecal microbiota of growing rabbits, as evidenced by non-significant changes in α and β diversity. However, differential analysis demonstrated that 0.3 g/kg XOS significantly increased the relative abundance of *Ruminococcaceae_UCG-013*, *Anaeroplasma*, *Anaerovorax*, *Paraprevotella* and *Coprococcus 2*. Conversely, 0.4 g/kg XOS notably reduced *Shuttleworthia* abundance, while 0.5 g/kg XOS decreased *Ruminococcaceae_UCG-010*, *Butyricimonas*, *Ruminiclostridium 1*, *Family XIII AD3011 group* and *Faecalibaculum*. Notably, *Ruminococcaceae_UCG-013* has been identified as a potential biomarker for obesity alleviation [57], and is associated with various fatty liver diseases in some studies [58, 59]. *Paraprevotella* may enhance host defense through its type IX secretion system, promoting trypsin autolysis to protect IgA from degradation and inhibiting pathogens like mouse hepatitis virus-2 [60]. *Coprococcus 2* is functionally linked to carbohydrate fermentation [61, 62]. These findings suggest that XOS might influence carbohydrate metabolism, enzymatic activity, and pathogen resistance by modulating specific bacterial taxa including *Coprococcus 2*, *Paraprevotella*, *Ruminococcaceae_UCG-013* and *Shuttleworthia*. Intriguingly, the glycolytic capacity of *Shuttleworthia* for producing acetate, butyrate, and lactate [63] showed a significant correlation with valeric acid levels in our study. Both 0.4 and 0.5 g/kg XOS treatments significantly increased valeric acid content, implying XOS may enhance valeric acid production by altering *Shuttleworthia's* metabolic activity. While our observations reveal XOS-induced bacterial differences, current data limitations permit only partial understanding of these changes, further investigations are required to elucidate their broader biological implications in gut homeostasis, disease intervention and animal husbandry applications.

## Conclusion

Our findings demonstrate that XOS enhances feed consumption in rabbits, which may be attributed to improved fiber digestibility via bacterial modulation and enhanced valeric acid production. Furthermore, XOS supplementation exhibited a protective effect against oxidative stress induced by dietary challenges and potentially enhanced resistance to intestinal pathogen colonization. These combined effects highlight the potential of XOS as a multifunctional dietary supplement in rabbit farming. However, further investigations are warranted to fully elucidate the complex interactions between the microbiota and the host.

## Supplementary Information

Additional file 1: Linear and quadratic analysis of XOS on growth performance in growing rabbits.

## Data Availability

The data used to support the findings of this study are available from the corresponding author on reasonable request.

## Abbreviations

ADF: Acid detergent fiber
ADFI: Average daily feed intake
ADG: Average daily gain
ADL: Acid detergent lignin
AIA: Acid insoluble ash
ALB: Albumin
ALP: Alkaline phosphatase
ALT: Alanine aminotransferase
AST: Aspartate aminotransferase
CF: Crude fiber
CP: Crude protein
DM: Dry matter
EE: Ether extract
FCR: Feed conversion rate
GE: Gross energy
GLB: Globulin
GLU: Glucose
GSH-Px: Glutathione peroxidase
IgA: Immunoglobulin A
IgG: Immunoglobulin G
IgM: Immunoglobulin M
MDA: Malondialdehyde
MUC 2: Mucin 2
NDF: Neutral detergent fiber
SCFA: Short chain fatty acid
sIgA: Secretory immunoglobulin A
SOD: Superoxide dismutase
TC: Total cholesterol
TG: Triglyceride
TP: Total protein
WGR: Weight gain rate
XOS: Xylooligosaccharides
