# Correction: Dietary xylooligosaccharides modulate oxidative stress and pathogen resistance in growing rabbits

**DOI:** 10.1186/s40104-025-01344-0

**Published:** 2026-02-12

**Authors:** Aipeng Mao, Xiaoyan Peng, Junning Pu, Yanbin Chen, Qingyue Liu, Jingyi Cai, Hua Zhao, Gang Jia, Gang Tian

**Affiliations:** 1 Animal Nutrition Institute, Sichuan Agricultural University, Chengdu, Sichuan 611130 People’s Republic of China; 2 Key Laboratory of Animal Disease‑resistant Nutrition, Ministry of Education, Ministry of Agriculture and Rural Affairs, Key Laboratory of Sichuan Province, Chengdu, Sichuan 611130 People’s Republic of China

**Correction: J Animal Sci Biotechnol 16, 145 (2025)**

**https://doi.org/10.1186/s40104-025-01268-9**

Following publication of the original article [1], the authors found an error in Fig. 5 and its caption, in which "ileum" should be corrected to "jejunum". Fig. 5 has been corrected from:

**Fig. 5:**
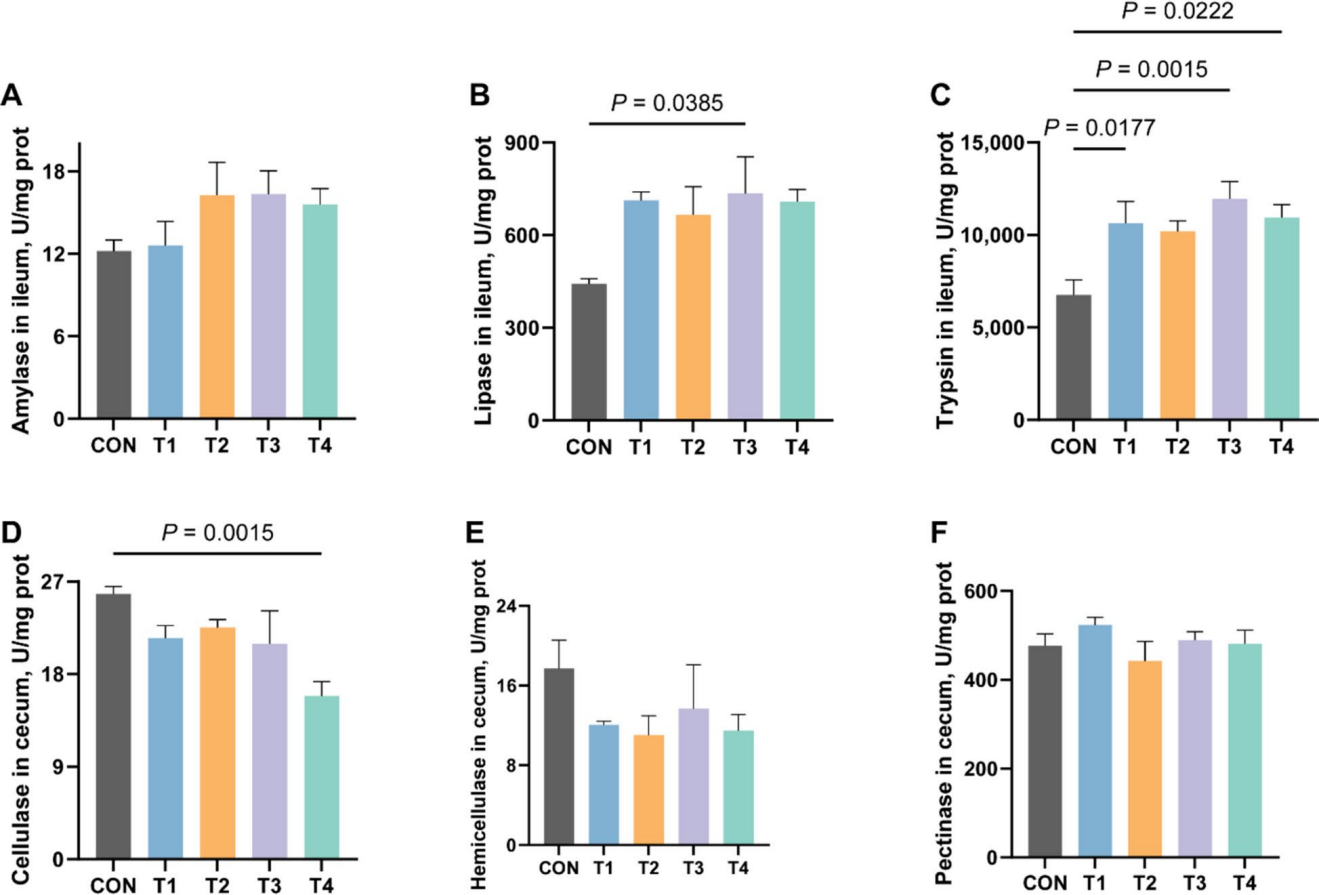
Effects of XOS on the activity of digestive enzymes in growing rabbits. **A** The activity of amylase in the ileum of growing rabbits. **B** The activity of lipase in the ileum of growing rabbits. **C** The activity of trypsin in the jejunum of growing rabbits. **D** The activity of cellulase in the caecum of growing rabbits. **E** The activity of hemicellulose in the caecum of growing rabbits. **F** The activity of pectinase in the caecum of growing rabbits

To:

**Fig. 5:**
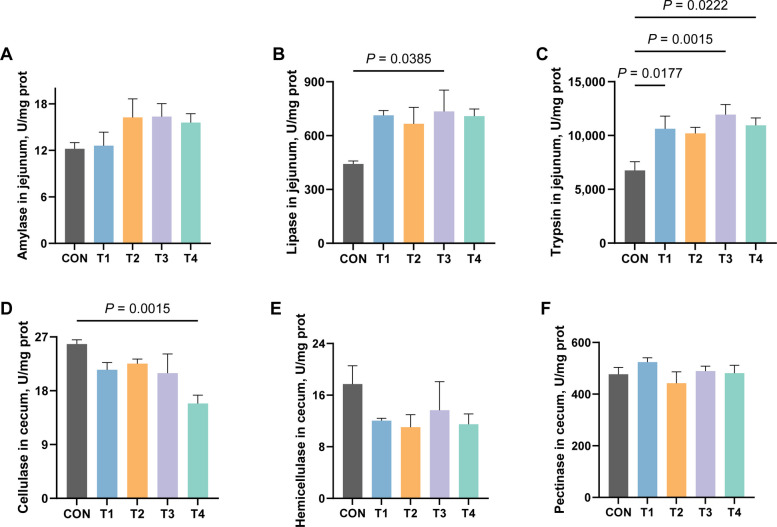
Effects of XOS on the activity of digestive enzymes in growing rabbits. **A** The activity of amylase in the jejunum of growing rabbits. **B** The activity of lipase in the jejunum of growing rabbits. **C** The activity of trypsin in the jejunum of growing rabbits. **D** The activity of cellulase in the caecum of growing rabbits. **E** The activity of hemicellulose in the caecum of growing rabbits. **F** The activity of pectinase in the caecum of growing rabbits

The original article [1] has been updated.
