# Supplementary material for: Dietary xylooligosaccharides modulate oxidative stress and pathogen resistance in growing rabbits
Source: J Anim Sci Biotechnol. 2025 Nov 7;16:145. doi: 10.1186/s40104-025-01268-9 (PMC12593894; doi:10.1186/s40104-025-01268-9)
Supplement: Supplementary file 1 — Additional file 1: Linear and quadratic analysis of XOS on growth performance in growing rabbits. [file 40104_2025_1268_MOESM1_ESM.docx]

**Supplemental table 1 Linear and quadratic analysis of XOS on growth performance in growing rabbits**

| **Items** | **Groups** | | | | | ***P-*value** | |
| --- | --- | --- | --- | --- | --- | --- | --- |
|  | **CON** | **T1** | **T2** | **T3** | **T4** | **Linear** | **Quadratic** |
| **Initial BW, g** | 890.21±7.84 | 888.21±7.71 | 895.37±7.58 | 889.85±7.84 | 887.59±7.71 | 0.881 | 0.885 |
| **BW on d 21, g** | 1502.33±18.42 | 1522.77±17.69 | 1488.48±18.04 | 1468.56±18.04 | 1466.52±17.36 | 0.025 | 0.075 |
| **Final BW, g** | 1953.14±35.65 | 1994.00±31.44 | 1978.00±32.36 | 1870.60±29.83 | 1910.21±30.60 | 0.032 | 0.085 |
| **D 1 to 21 (n=30)** | | | | | |  |  |
| **ADG, g/d** | 28.86±0.88 | 30.18±0.82 | 27.17±0.88 | 27.60±0.84 | 27.69±0.79 | 0.063 | 0.173 |
| **WGR** | 0.69±0.02 | 0.72±0.02 | 0.63±0.02 | 0.65±0.02 | 0.66±0.02 | 0.049 | 0.104 |
| **ADFI, g/d** | 81.06±1.14 | 85.43±1.12 | 84.06±1.14 | 84.29±1.14 | 84.77±1.12 | 0.090 | 0.084 |
| **FCR** | 2.84±0.06 | 2.89±0.06 | 3.07±0.08 | 3.02±0.06 | 3.12±0.06 | ＜0.001 | 0.002 |
| **D 21 to 35 (n=30)** | | | | | |  |  |
| **ADG, g/d** | 33.84±1.64 | 33.93±1.44 | 33.92±1.49 | 29.73±1.37 | 33.09±1.41 | 0.220 | 0.403 |
| **WGR** | 0.54±0.03 | 0.54±0.02 | 0.54±0.02 | 0.47±0.02 | 0.51±0.02 | 0.072 | 0.180 |
| **ADFI, g/d** | 109.10±2.63 | 116.53±2.32 | 115.11±2.38 | 110.90±2.20 | 115.64±2.25 | 0.460 | 0.593 |
| **FCR** | 3.28±0.13 | 3.49±0.11 | 3.46±0.12 | 3.64±0.11 | 3.54±0.11 | 0.084 | 0.143 |
| **D 1 to 35 (n=30)** | | | | | |  |  |
| **ADG, g/d** | 30.17±1.00 | 31.64±0.88 | 30.30±0.94 | 27.84±0.84 | 29.18±0.86 | 0.036 | 0.110 |
| **WGR** | 1.23±0.04 | 1.24±0.04 | 1.19±0.04 | 1.10±0.04 | 1.14±0.04 | 0.011 | 0.039 |
| **ADFI, g/d** | 90.03±1.91 | 97.76±1.75 | 96.62±1.80 | 93.76±1.66 | 96.19±1.70 | 0.240 | 0.134 |
| **FCR** | 3.09±0.09 | 3.09±0.08 | 3.19±0.08 | 3.48±0.07 | 3.33±0.07 | ＜0.001 | 0.003 |
